# FUNC: a package for detecting significant associations between gene sets and ontological annotations

**DOI:** 10.1186/1471-2105-8-41

**Published:** 2007-02-06

**Authors:** Kay Prüfer, Bjoern Muetzel, Hong-Hai Do, Gunter Weiss, Philipp Khaitovich, Erhard Rahm, Svante Pääbo, Michael Lachmann, Wolfgang Enard

**Affiliations:** 1 Max-Planck-Institute for Evolutionary Anthropology, Deutscher Platz 6, D-04103 Leipzig, Germany; 2 Interdisciplinary Center for Bioinformatics, University of Leipzig, Härtelstr. 16-18, D-04107, Germany; 3 Institute for Computational Biology, Shanghai Institutes for Biological Sciences, Chinese Academy of Sciences, 320 Yue Yang Road, Shanghai, 200031, China

## Abstract

**Background:**

Genome-wide expression, sequence and association studies typically yield large sets of gene candidates, which must then be further analysed and interpreted. Information about these genes is increasingly being captured and organized in ontologies, such as the Gene Ontology. Relationships between the gene sets identified by experimental methods and biological knowledge can be made explicit and used in the interpretation of results. However, it is often difficult to assess the statistical significance of such analyses since many inter-dependent categories are tested simultaneously.

**Results:**

We developed the program package FUNC that includes and expands on currently available methods to identify significant associations between gene sets and ontological annotations. Implemented are several tests in particular well suited for genome wide sequence comparisons, estimates of the family-wise error rate, the false discovery rate, a sensitive estimator of the global significance of the results and an algorithm to reduce the complexity of the results.

**Conclusion:**

FUNC is a versatile and useful tool for the analysis of genome-wide data. It is freely available under the GPL license and also accessible via a web service.

## Background

High-throughput genomic technologies are revolutionizing biology and medicine and provide new challenges in the way we analyse and interpret these large amounts of data. To this end it is necessary to integrate the acquired knowledge into a flexible data structure and the Gene Ontology (GO) Consortium has provided a widely used solution to this challenge by describing properties of genes using a controlled vocabulary and representing them in a directed acyclic graph, which groups genes in categories [1]. Consequently, there has been an explosion in the number of methods to investigate large-scale gene expression data in the context of these functional annotations. The principle underlying most of these methods is the identification of an enrichment in particular gene annotations among a selected set of genes compared to a reference set – for example, an enrichment of differentially expressed genes in certain annotation categories compared to all other genes on a microarray. The significance of the enrichment is tested for example using tests based on the hypergeometric or the binomial distribution, Fisher's exact test, or a chi-square test. Various programs have implemented this approach (e.g. [2-7]). Ben-Shaul and others have argued that in many cases such a discrete distinction between "differentially expressed" and "not differentially expressed" genes is arbitrary and can reduce the power of the study to identify enriched categories (e.g. [8]). Therefore, methods have been proposed that instead use a measure of choice to rank the genes, and then use rank-based tests such as the Wilcoxon-rank test or the Kolmogorov-Smirnov test to test if genes that belong to one category differ in their ranks from genes that do not belong to the category (e.g. [8]). In both cases – rank-based classification or discrete categorization – and independently of the statistic that is used to define the significance of a single category, one needs to correct for the large number of tests performed in such an analysis. This is a challenging task, since the tested categories are highly inter-dependent: a single gene is usually annotated in many categories, and categories subsume other categories. One approach for the correction is to compute the family-wise error rate (FWER), i.e. to estimate the probability that at least one false positive category exists among those that are labelled significant. Applications exist that use a simple Bonferroni correction or other more powerful FWER correction procedures [9-11]. However, it has been noted that controlling the FWER might be too conservative in many genomic applications and that it might instead be more useful to determine the false discovery rate (FDR) [12], i.e. the proportion of false positives among all significant features, since a known proportion of false positives can easily be tolerated for an increase of power in these contexts (see e.g. [13] for an overview). Several approaches to estimate the FDR have been proposed (see e.g. [14,15]) and some have been integrated in various functional profiling applications (e.g. [5,9,16-21]). Many methods rely on permutations to estimate the FWER or FDR, since the dependency and structure of the annotations makes it difficult to find analytical methods to do the same [22]. Usually the gene lists are permuted, though some approaches also allow the permutation of sample labels [16,20,21,23], This allows not only a correction of dependence among the categories, but also for dependence among the genes [23].

In this paper we present the program package called FUNC, which includes and extends on the methods described above: It allows selecting among four different kinds of tests, depending on the type of data to be analysed (see also Table 1): (i) a test based on the hypergeometric distribution for analyzing binary associated variables (e.g. differentially expressed, not differentially expressed), (ii) a Wilcoxon rank test for a continuous associated variable (e.g. probability of being differentially expressed), (iii) a binomial test to compare the ratio of two counts per gene in (e.g. human amino acid changes versus chimpanzee amino acid changes) and (iv) a 2 × 2 table test that is suitable for a McDonald Kreitman type test to infer selection on genes from divergence and polymorphism data at two types of sites, like synonymous and non-synonymous sites in a coding sequence [24]. The two latter methods have not previously been implemented in any GO statistical analysis application, and should be especially useful for analyzing genome-wide DNA sequences. These approaches, as implemented in FUNC, have already been used for the analysis of the chimpanzee genome sequence [25]. FUNC also uses permutations of genes to calculate for each category, i.e. each p-value cut-off, a FWER and a FDR estimate. In addition, FUNC provides a global test statistic to gauge the significance of the complete data set, which has not been implemented in other programs to date. The global test statistic tests whether the complete distribution of functional annotations differs from a random distribution and in this way allows determining an overall significance for the data set. Another method implemented in FUNC is the ability to refine the results by eliminating extraneous categories marked as significant. Some categories are significant solely due to the fact that their subtree includes categories that are significant. Thus, their significance does not provide additional information beyond that of their descendant categories and their exclusion from lists of significant categories can be helpful for interpreting and representing the results. In summary, FUNC provides a useful and sensitive tool to analyse annotations in the context of a variety of genomic data.

**Table 1:** Properties of the four category tests

**Test**	**Gene associated variable**	**Alternative hypothesis (one side)**	**Alternative hypothesis (other side)**	**Example**
Hypergeometric	binary	Category contains lower proportion of variable "1" as the root category	Category contains higher proportion of variable "1" as the root category	Gene list with detected genes on an array; "0" is not differently expressed, "1" is differently expressed
Wilcoxon rank	continuous	Sum of ranks of genes in the category is higher than all other genes	Sum of ranks of genes in the category is lower than all other genes	Gene list with detected genes on an array; continuous variable is the probability for being not differently expressed
Binominal	two counts	Frequency of countA in category is lower than in root category	Frequency of countB in category is lower than in root category	CountA is amount of SAGE tags in experiment A, countB is amount of SAGE tags in experiment B
2 × 2 contingency	four counts	Counts are dependent and countA/countB < countC/countD	Counts are dependent and countA/countB > countC/countD	CountA and countC are differences at nonsynonymous sites between and within species, countB and countD are differences at synonymous sites between and within species, respectively

## Implementation

### Overview

**Figure 1:**
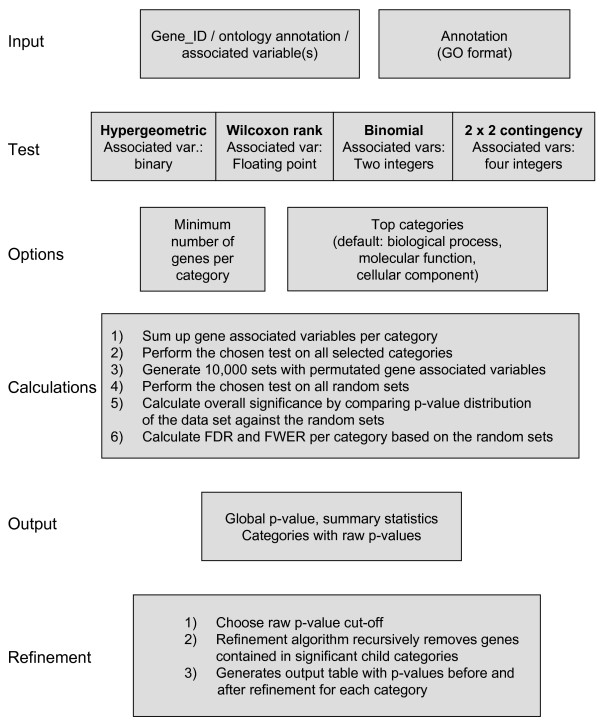
**Schematic overview of FUNC**. See main text for a description.

FUNC is a set of four command line tools that allow the analysis of a set of genes with respect to their annotation (see Figure 1 for a schematic overview). It is particular useful when analyzing ontological annotations such as provided by the Gene Ontology consortium [1] or eVOC [26], but can be easily adapted to any other annotation. Each of the command line tools performs a specific statistical test on a certain type of input data. The user can select the ontology or subtree of an ontology on which the test should be performed, and restrict the tested categories to those containing some minimum number of genes. For each category in the ontology the statistical test of the used tool is performed resulting in the "raw p-value" for that category. Since many categories are tested and the tests are inter-dependent in a complex manner, FUNC compares the test results to results obtained from random datasets in which the gene-associated variables are permuted. This specifies the null hypotheses, namely that there is independency between the associated variable and the annotation of the genes. These random sets are used to calculate for each category, i.e. each raw p-value cut-off, two corrected p-values: a resampling-based false discovery rate (FDR) [27] and the family-wise error rate (FWER) [28]. The FDR is an estimate of the proportion of categories that are false positives among all the categories with a raw p-value equal or lower than the given category. The FWER is the estimated probability that at least one false positive category exists among all the categories with a raw p-value equal or lower than the given category. In addition, FUNC compares the distribution of raw p-values of the random sets with the distribution of raw p-values of the data set in order to obtain an overall significance p-value – a Kolmogorov-Smirnov-type test against a single null hypothesis stating that the gene associated variables are randomly distributed across all the categories. This global test statistic is useful since it is expected to be sensitive even if a weak signal is distributed among many categories. The overall significance value can be used to decide whether the data set is at all differently distributed from random. If this is the case, one can then pick a FDR to decide which of the categories deviate significantly. This procedure might be preferable to a-priori selection of an FDR or FWER significance level or changing them post-hoc.

The output of FUNC is a summary of the above-mentioned statistics as well as a table listing the analysed categories and the associated raw and corrected p-values. After picking a desirable p-value cut-off, the user can run the refinement algorithm to identify those significant categories that provide the most concise information, i.e. to identify those categories whose significance does not depend solely on significant descendant categories.

### Category tests

Each of the four FUNC tools is designed for one of the possible category tests: hypergeometric test, Wilcoxon rank test, binomial test and 2 × 2 test (Table 1). For each test, two p-values for both sides of the test statistic are calculated, which allows the detection of an enrichment or a depletion of gene-associated variables among the categories. A detailed description of the algorithms used can be found in the Supplement. Briefly, the hypergeometric test takes a binary variable (e.g. "0" and "1") that is associated with each gene (e.g. "1" for differentially expressed and "0" for equally expressed) and uses the hypergeometric distribution to calculate for each category the probability to "draw" this many or more (respectively this many or less) differently expressed genes from the top category of the subtree selected by the user.

The Wilcoxon rank test differs from this scheme only in that it takes a floating point variable instead of a binary variable and compares the ranks of the genes in the tested category with the ranks of the remaining genes in the top category. This test is useful when it is not possible to clearly classify genes to two distinct classes – as is often the case in microarray experiments. This kind of test has also been used previously in the comparison between the human and the chimpanzee genome to identify GO categories, which contain an excess of fast or slowly evolving genes [25].

Whereas the hypergeometric and Wilcoxon rank tests compare the distribution of one gene-associated variable among categories, the "binomial test" compares the ratio of two gene-associated counts among categories. Each gene is associated with two counts, and the test determines whether the ratio of these counts in a category is significantly different from the ratio in the top category. The binomial test has been used to identify categories containing more amino acid changes on the human lineage compared to the number of changes on the chimpanzee lineage, and to identify categories that have a higher than expected number of amino acid changes between human and chimpanzee compared to changes between mouse and rat [25](see also the example below). This test might also be useful when comparing counts of expressed sequence tags e.g. from two SAGE (Serial Analysis of Gene Expression) libraries [29].

The fourth test takes a 2 × 2 table as the associated gene variables, sums them over each category, and uses a Fisher's exact test or a chi-square test (if all values in the tested category are larger then ten) to test whether the two properties (in rows and columns, each in two states) are independent of each other. Note that in contrast to the other three tests, the calculated p-value is not dependent on an expectation taken from the top category. This test can be useful to conduct a McDonald-Kreitman type test [24] on GO categories. A McDonald-Kreitman type of test compares the number of fixed substitutions and the number of polymorphisms at two classes of sites, such as synonymous and non-synonymous sites. An excess of fixed non-synonymous substitutions can indicate the action of positive selection, whereas an excess of non-synonymous polymorphisms can indicate the presence of slightly deleterious amino acid variants (for a review see [30]). The 2 × 2 contingency table test implemented in FUNC calculates two separate p-values to test for an excess of non-synonymous substitutions and an excess of non-synonymous polymorphisms, respectively [see Additional file 1]. The availability of a large (and largely unbiased) genome-wide measurement of polymorphisms in humans and other species together with the already available data on substitutions should make this test very useful in the near future.

It is important to keep in mind that for all the four tests the power to reject the null hypothesis differs among different categories since categories differ in their amount of genes and/or their amount of gene associated counts. Hence, the category with the biggest size effect is not necessarily the most significant category and vice versa (see also [31] and reply). Also note, for the binomial test and the 2 × 2 contingency table test, that the null hypothesis that FUNC tests is that the genes and not the gene associated counts are a random sample in a category. As a consequence the raw p-values of these two tests should be considered more like an arbitrary test statistic which is compared to the distribution of p-values obtained by permuting genes and not single gene counts (see also example below).

### Correction for multiple testing

When many hypotheses are tested at the same time it is expected that a number will appear significant even if all the null hypotheses hold. Therefore, in order to confidently reject some of the null hypotheses, it is necessary to correct for multiple testing. The types of large-scale genomic experiments that have become possible during recent years, in particular microarrays, have revived interest in different statistical methods that deal with the issue of multiple testing (e.g. [13,15]). The issue is somewhat complex, since (1) the tests are interdependent in a complex manner, (2) the power of each single test is often low, (3) more than one of the tested hypotheses are usually truly not null and (4) rejected hypotheses can be regarded as candidates for additional tests, so that less conservative significance levels can be tolerated for an increase in power. All of these issues are also relevant in the context described here, in particular the complex interdependency of the tests. To overcome the interdependency, we chose to use permutations, i.e. the randomization of gene-associated variables, in order to model the distribution under the null hypothesis that gene associated variables are independent of the gene annotation. This permuted data, the random sets, can be used to estimate the family wise error rate, i.e. the probability that among the tests that are declared significant one or more are false positives [28]. This approach is more powerful than the conservative Bonferroni correction and has, in the context of GO analyses programs, been implemented for example in FuncAssociate [10] and is also implemented in FUNC. For any of the four tests described above one can calculate a raw p-value cut-off for which the FWER is e.g. 5%. This approach is, however, very strict, and a certain (known) fraction of false positives among the significantly labelled tests can often be tolerated for an increase of power. This is the reason why the false discovery rate (FDR) has gained popularity within the genomic community. The FDR is (loosely) defined as the expected proportion of falsely rejected hypothesis among all rejected hypotheses. Different methods exist to estimate the FDR, differing in how they treat the case that no hypothesis is rejected and in how they estimate the number of falsely rejected hypotheses (see e.g. [14,15,32] for a discussion). Several programs that analyse functional annotations have implemented FDR methods (e.g. [5,9,16-21]), most often using the procedure of Benjamini and Hochberg [12]. In FUNC we implemented a similar method by Yekueteli and Benjamini that is well-suited for resampling methods [27]. Although it has been shown that the method also works well under positive dependency among the tests [33] and an easy (conservative) correction method can be used under different kinds of dependencies [33], it is not entirely clear whether it is well-suited for the kind of strong dependencies that exist among the functional annotations. Further, it is noteworthy that the current methods for computing the FDR are strictly valid only under the assumption of subset pivotality, i.e. under the assumption that the significance of one test does not depend on the significance of another test. However, this assumption is violated for the hypergeometric test, the Wilcoxon rank sum test and the binomial test, since the expectation for each category is derived from the top category, which includes the other categories tested. Hence, if one or more categories truly deviate from the null hypothesis, this influences the null expectation of other categories. However, there is often no reasonable alternative for estimating the expectations independently. In practice, all these issues will not be relevant for data sets which deviate considerably from a random functional annotation. But for data sets with a weak signal, the FDR might not qualify as a well-suited measurement to determine whether there is any (or no) indication for a non-random distribution of the gene-associated variables among functional categories. Therefore, in addition to estimating the FDR and FWER rate of the data set, we developed a method to test directly the "global null hypothesis" that gene-associated variables are randomly distributed among categories.

### Testing the global null hypothesis

**Figure 2:**
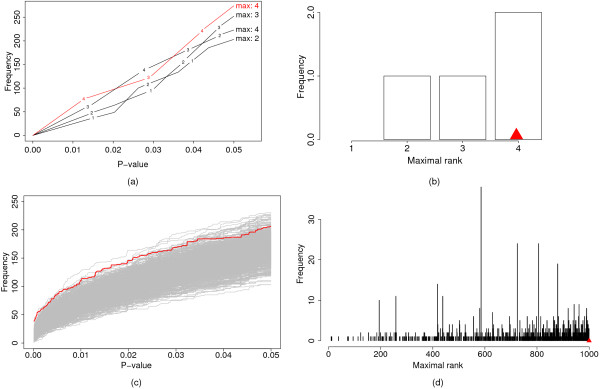
**Illustration how the global p-value is calculated**. On the left ((a) and (c)) the cumulative p-value distribution between 0 and 0.05 is shown for the data set (red line) and the random sets (black or gray lines). For each distribution its maximal rank is determined and the maximal rank of the data set (red arrow) is compared to the maximal ranks of the random sets ((b) and (d)). The upper two panels exemplify this principle with three random sets and the lower two panels show the result of testing the ontology molecular function for an excess of amino acid changes in primates (see results and discussion).

As was pointed out above, when the strength of the deviation may be weak, it is useful to test whether the data shows any deviation from the random distribution before embarking on finding out which categories deviate from others. For this we calculate a p-value to test the null hypothesis that the gene-associated variables are randomly distributed among all categories. This is done by looking over all possible cut-offs of the raw p-values between 0 and 0.05, and for each of them calculating the proportion of random sets that have as many or more categories showing this many or fewer raw p-values. Then, in a similar fashion to the Kolmogorov-Smirnov test, we find the cut-off value for which the deviation from the random sets is maximal. We then do the same test (finding the cut-off with maximal distance) to every one of the random sets. The overall p-value is then determined by calculating the proportion of random sets that have the same or a larger maximal distance (see Additional file 1 and Figure 2). If this p-value is low (e.g. smaller than 0.05), one can reject the null hypothesis that the gene-associated variable(s) are distributed independently of their functional annotations.

This measure should be especially helpful, when the signal is weak and/or is distributed among many categories and should be more sensitive than an FDR estimate (see example below). The FDR can be interpreted using an analogy of how much money one would be willing to waste. Testing the global null hypothesis can determine whether it is worth spending any money in the first place, and the FDR can subsequently be used to estimate how much money one is willing to waste.

### Refinement

**Figure 3:**
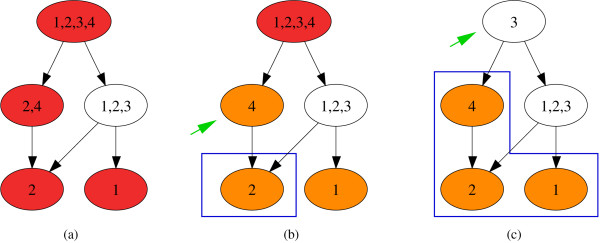
**Illustration of the refinement algorithm**. (a) Before the refinement, four groups are labelled significant (red) that contain the genes 1–4. (b) On the deepest level of the tree significant categories remain significant (orange). On the next level a significant category (arrow) is tested after all genes in the significant descendant categories (blue box) are removed. In this example, the category remains significant. (c) This procedure is repeated for the category on the next level (arrow) and again all genes in significant descendant categories (blue box) are removed. In this example the category is no longer significant after refinement.

Once one is confident that categories showing enrichment or depletion of the gene-associated variable(s) exist, and after choosing a suitable raw p-value as a cut-off, based on a certain FDR or FWER, it is useful to specify the deviation as precisely as possible. This means one wants to exclude categories that are significant solely because they contain significant descendant categories. The refinement algorithm starts from the leaves (i.e. the most specific annotation), recursively removes the genes annotated in significant descendant categories, and tests the remaining genes in a significant parent category again (Figure 3). In this way the list of all significant categories can be limited to the most specific categories, which make the results more interpretable and manageable. This algorithm is similar to the *elim *algorithm proposed recently [34]. However, in contrast to the *elim *or the related *weight *algorithm [34] we interpret the results of the refinement like a *post hoc *test. Consider a hypothetical example where the gene associated variables are significantly overrepresented in the category carbohydrate metabolism, which is due to an overrepresentation in the two descendant categories glycolysis and tricarboxylic acid cycle. It is true regardless of the refinement that genes annotated in carbohydrate metabolism are overrepresented in the data set. That genes annotated in glycolysis and tricarboxylic acid cycle are overrepresented is just the more specific statement. We find it helpful and transparent to distinguish between significant categories and the most specific significant categories and hence find it useful to separate these two analyses.

## Results and discussion

To demonstrate the usefulness of FUNC we analysed a dataset of 7034 orthologous genes compared between human, chimpanzee, mouse and rat [25]. We asked whether there are GO categories that evolve faster than expected in either rodents or primates. For this purpose we added the number of amino acid changes for a given gene between mouse and rat (rodent) and human and chimpanzee (primate) and performed the binomial test described above [see Additional file 2]. This procedure treats all genes in a category essentially as a single gene. Note that the p-value of this test is only nominal since it assumes independency among amino acid substitutions, but that the global p-value, the FWER and the FDR calculated by FUNC is based on the permutations across genes and hence controls for the dependency of amino acid substitutions within genes. For brevity, we limit the analysis here to the results from the ontology molecular function.

Of the 7043 genes, 4303 genes have an annotation in the ontology molecular function (Table 2). The binomial test is two-sided, i.e. for each category it tests whether there are more amino acid changes in primates and tests whether there are more amino acid changes in rodents. The expected value is given by the ratio of amino acid changes of all genes annotated in the ontology molecular function. The global p-value is 0.0019 and 0.0008 for rodents and primates, respectively, since in the 10,000 permutations performed only 19 and 8 sets, respectively, had maximal ranks of p-values that were equal or higher than for the data set (Fig. 2). This indicates that categories exist, which evolve faster in primates than in rodents and faster in rodents than in primates. This is not due to different mutation rates in rodents and primates among the different categories since changes at silent sites do not show such a significant grouping when tested accordingly (data not shown). Interestingly, none of the categories that evolve faster in primates had a FDR or FWER estimate below 0.05, exemplifying that the global p-value is more sensitive in detecting a deviation from the null hypothesis than FDR or FWER estimates for single categories. At a FDR threshold of 0.2, 38 categories evolve faster in primates. In order to get the categories with the most specific annotation, we ran the refinement algorithm at the corresponding raw p-value, which resulted in 13 categories (Table 2). These categories might evolve faster in primates because they experienced more positive selection in primates than in rodents or because they evolved under less constraint in primates than in rodents (see e.g. [25] for a discussion). Olfactory receptors, which are also identified here (Table 2), are thought to have evolved under less constraint in primates than in rodents since a higher fraction of pseudogenes is found in primates [35], indicating that the more sensitive global test statistic identified biologically relevant categories in the analysed example.

**Table 2:** Categories evolving fast in humans and chimpanzees

**GO ID**	**Category**	**Genes^a^**	**AA rod.^b^**	**AA prim.^c^**	**Ratio^d^**
GO:0003674	molecular_function^e^	4303	84306	8604	0.1
GO:0004889	nicotinic acetylcholine-activated cation-selective channel activity	6	85	29	0.34
GO:0004984	olfactory receptor activity	5	78	32	0.41
GO:0005184	neuropeptide hormone activity	13	95	31	0.33
GO:0005217	intracellular ligand-gated ion channel activity	2	117	30	0.26
GO:0005272	sodium channel activity	9	124	34	0.27
GO:0005279	amino acid-polyamine transporter activity	19	276	63	0.23
GO:0005523	tropomyosin binding	4	14	12	0.86
GO:0008188	neuropeptide receptor activity	17	212	44	0.21
GO:0008271	sulphate porter activity	4	107	37	0.35
GO:0015194	L-serine transporter activity	1	7	8	1.14
GO:0016652	oxidoreductase activity, acting on NADH or NADPH, NAD or NADP as acceptor	10	140	36	0.26
GO:0031402	sodium ion binding	31	684	109	0.16
GO:0031404	chloride ion binding	26	459	79	0.17

^a ^number of genes analysed in category; ^b ^number of amino acid changes between mouse and rat; ^b ^number of amino acid changes between human and chimpanzee; ^b ^number of amino acid changes between mouse and rat; ^**d**^ratio of primates/rodents; ^**e **^the ratio in the top category, in this case molecular function, gives the expected ratio.

## Conclusion

We present the software package FUNC, which enhances the ability of researchers to correlate their data with gene annotations that are often provided in the form of ontologies. FUNC currently has two main drawbacks. First, it does not provide any graphical representation of the results such as those provided e.g. by GOMiner [36]. Second, it allows no easy way to permute sample-associated variables instead of gene-associated variables. This has been shown to be useful in some cases [23] and has been implemented by some programs [16,20,23]. However, despite these two drawbacks, FUNC provides considerable advantages over existing tools: it integrates four kinds of tests suitable for the analysis of gene expression data and DNA sequence data of which two (binomial test on two gene-associated counts and 2 × 2 contingency table test on four gene-associated counts) are not implemented in other GO analysis programs. FUNC also provides two established multiple correction methods (FDR and FWER) as well as a new overall significance estimate, which should be useful especially for data with a weak signal. Further, the implemented refinement algorithm is a useful and transparent method for extracting the most specific biological information from lists of significant GO categories. Finally, FUNC is available as a well-documented stand-alone tool for UNIX/GNU Linux platforms as well as accessible via a web service, which makes its use more flexible than many other available GO analysis tools. Thus, FUNC provides flexible, statistically rigorous and novel tools to analyse the functional annotation of a variety of genome-wide data.

## Availability and requirements

**Project name: **FUNC

**Project home page: **

**Operating System: **Unix/GNU Linux

**Programming languages: **C++, Perl, bash

**Other requirements: **R mathematical library (libRmath)

**License: **GNU GPL V2.0

## Authors' contributions

KP developed and wrote the software, BM, HHD, GW and PK contributed to and conceived earlier versions of the software, ER and SP provided resources, ML conceived the statistical analyses, WE conceived the study and wrote the manuscript. All authors read and approved the manuscript.

## Supplementary Material

Additional file 1 description of used methods and algorithms Click here for file

Additional file 2 Input file used for comparing amino acid changes between primates and rodents Click here for file
