# Supplementary material for: FUNC: a package for detecting significant associations between gene sets and ontological annotations
Source: BMC Bioinformatics. 2007 Feb 6;8:41. doi: 10.1186/1471-2105-8-41 (PMC1800870; doi:10.1186/1471-2105-8-41)
Supplement: Additional file 1 — description of used methods and algorithms [file 1471-2105-8-41-S1.doc]

## Methods and Algorithms

### Input data

FUNC requires two types of information; a file containing information about the structure of the ontology, and a file containing the annotations and data to be tested.

(i) The ontology structure files

FUNC uses three files from the termdb-tables distribution provided by the Gene Ontology Consortium (see http://archive.godatabase.org/full/) to read the structure of Gene Ontology. A reformatted version of eVoc [1] suitable for use in FUNC is available via the FUNC-website.

(ii) Input data files

The input file contains annotations and data for the test to be performed in a simple tab separated values format (see website for a detailed description). Gene annotations for gene sets to be tested can be obtained from the GO Website (<http://www.geneontology.org/GO.downloads.shtml>). Tools like EnsMart [2] or GenMapper [3] can be used by researchers to obtain GO annotations for genes which do not have identifiers with direct links to GO.

### Hypergeometric test

When the top category of the tested subtree contains *N* genes and *A* of them have the attribute of interest, the p-value of randomly drawing *a* or more genes with this attribute among *n* genes within a category is calculated by:

$\sum_{i=a}^{n} \frac{\left(\begin{matrix} A \\ i \end{matrix}\right)\left(\begin{matrix} N-A \\ n-i \end{matrix}\right)}{\left(\begin{matrix} N \\ n \end{matrix}\right)}$.

The p-value for randomly drawing *a* or less genes with this attribute among *n* genes within a category is calculated by:

$$\sum_{i=0}^{a} \frac{\left(\begin{matrix} A \\ i \end{matrix}\right)\left(\begin{matrix} N-A \\ n-i \end{matrix}\right)}{\left(\begin{matrix} N \\ n \end{matrix}\right)}$$

using the same parameters as above.

### Wilcoxon rank sum test

We perform a standard Wilcoxon rank sum test (following closely the implementation of the wilcox.test function of the R Statistical Language [4]). The ranks of the genes belonging to a tested category are compared to those in the top category not belonging to the tested category. Two p-values are calculated for an excess of high or low ranking genes in this category, respectively.

### Binomial test

Let *A* and *B* denote the sum of all gene associated variables within the top category of the tested subtree, and *a* and *b* the sums of these numbers in a category. First, the expectation for the fraction of *A* is calculated as *f=A/(A+B)*. Then

$\sum_{i=a}^{a+b} \left(\begin{matrix} a+b \\ i \end{matrix}\right)f^{i}\left(1-f\right)^{a+b-\text{i}}$and $\sum_{i=b}^{a+b} \left(\begin{matrix} a+b \\ i \end{matrix}\right)\left(1-f\right)^{i}f^{a+b-\text{i}}$ are used to calculate the p-value of having a number as high or higher than *a* respectively *b* in a category.

### 2x2 contingency test

Let *a*, *b*, *c* and *d* be the sum of the four gene associated variables in a category (representing e.g. the number of fixed non-synonymous substitutions between species, the number of polymorphic non-synonymous sites within a species, the number of fixed synonymous substitutions between species and the number of synonymous variable sites within a species, respectively, in the case of a McDonald-Kreitman type of test [5]). The p-value under the null hypothesis of independence between the two properties (class of site (state non-synonymous and state synonymous) and class of variation (state fixed between species and state polymorphic with one species) of the contingency table is calculated by a Fisher’s exact test. If all four values are greater than ten, we use a chi-square test to approximate the p-value. Without loss of generality, we define two p-values, whereas the first is valid in the case *a*/*b* > *c*/*d* and the second is valid for *a*/*b* < *c*/*d* and the non-valid p-value is set to 1. In the context of a McDonald-Kreitman type of test as exemplified above, a significant first p-value indicates an excess of fixed non-synonymous substitutions, indicating positive selection, whereas a significant second p-value indicates an excess of polymorphic non-synonymous substitutions, potentially indicative of slightly deleterious variants segregating within a population [5].

### FWER

Based on the method presented by Westfall and Young [6] the algorithm starts by finding the lowest p-value among all categories in each random set (subsequently called *r_i_*). For a category with a raw p-value *p* the corrected FWER p-value is then computed using the formula

$$\frac{\text{number r}_{\text{i}}\leq p}{\text{number of random sets}}$$

.

### FDR

The FDR is taken from expression (9) in [7]: Given *n*(*p*), the number of categories with a raw p-value less or equal to *p*, and *m_j_*(*p*), the corresponding number for random set *j*, the FDR for a category with raw p-value *p* is

$$\frac{1}{N}\sum_{j} \frac{m_{j}\left(p\right)}{m_{j}\left(p\right)+n\left(p\right)-pn\left(1\right)}$$

whereas *N* is the number of random sets and *n*(1) is the total number of categories in the data set [7]. As in [7], a value of -1 is given if *n*(*p*) minus the 95% quantile of *m_j_*(*p*) is bigger or equal than *n*(1)**p*.

### Global test-statistic

The global p-value is calculated using the cumulative distribution functions of the raw p-values from the data set and all random sets (see also Figure 2). For each p-value between 0 and 0.05 (in steps of 0.0001) we rank all sets starting from the set having the fewest significant categories and ending at the set with the highest rank, i.e. the set having the most significant categories at this given p-value (we artificially restrict the range of p-values to 0.05, in order not to lose power in the less relevant parts of the cumulative p-value distribution). For each set the maximal rank is calculated and the global p-value is computed by comparing this maximal rank of the data set to the number of random sets with a higher or equal rank:

### Refinement

In order to find the most precise functional description of a significant result the refinement algorithm searches for the first leaf category (i.e. with a depth first search) with significant (as specified by the user) subcategories, removes the genes of the significant subcategories and tests whether the category is significant with the remaining genes. This is done recursively until no significant leaf categories are left.

### Example data set used

Table S24 from [8] contains all the data. The 7043 Ensembl gene identifiers were mapped to 6734 Ensembl genes in Ensembl 40 and their corresponding GO identifiers. The termdb-tables distribution 200603 was used to read the structure of Gene Ontology.

### References

1. Kelso J, Visagie J, Theiler G, Christoffels A, Bardien S, Smedley D, Otgaar D, Greyling G, Jongeneel CV, McCarthy MI, et al: **eVOC: a controlled vocabulary for unifying gene expression data**. *Genome Res* 2003, **13**:1222-30.

2. Kasprzyk A, Keefe D, Smedley D, London D, Spooner W, Melsopp C, Hammond M, Rocca-Serra P, Cox T, Birney E: **EnsMart: a generic system for fast and flexible access to biological data**. *Genome Res* 2004, **14**:160-9.

3. Do HH, Rahm E: **Flexible Integration of Molecular-biological Annotation Data: The GenMapper Approach**. In: *Proceedings 9 International Conference on Extending Database Technology*: Springer LNCS; 2004.

4. R Development Core Team: **R: A language and environment for statistical computing**. Vienna, Austria; 2006.

5. McDonald JH, Kreitman M: **Adaptive protein evolution at the Adh locus in Drosophila**. *Nature* 1991, **351**:652-4.

6. Westfall PH, Young SS: **P-value adjustments for multiple tests in multivariate binomial models**. *J Am Stat Ass* 1989, **84**:780-786.

7. Yekutieli D, Benjamini Y: **Resampling-based false discovery rate controlling multiple test procedures for correlated test statistics**. *J Stat Plan Infer* 1999, **82**:171-196.

8. The Chimpanzee Sequencing and Analysis Consortium: **Initial sequence of the chimpanzee genome and comparison with the human genome**. *Nature* 2005, **437**:69-87.
